# STAT5a Confers Doxorubicin Resistance to Breast Cancer by Regulating ABCB1

**DOI:** 10.3389/fonc.2021.697950

**Published:** 2021-07-15

**Authors:** Zhaoqing Li, Cong Chen, Lini Chen, Dengdi Hu, Xiqian Yang, Wenying Zhuo, Yongxia Chen, Jingjing Yang, Yulu Zhou, Misha Mao, Xun Zhang, Ling Xu, Siwei Ju, Jun Shen, Qinchuan Wang, Minjun Dong, Shuduo Xie, Qun Wei, Yunlu Jia, Jichun Zhou, Linbo Wang

**Affiliations:** ^1^ Cancer Institute (Key Laboratory of Cancer Prevention and Intervention, China National Ministry of Education), 2nd Affiliated Hospital, School of Medicine, Zhejiang University, Hangzhou, China; ^2^ Sir Run Run Shaw Hospital, Zhejiang University, Hangzhou, China; ^3^ Biomedical Research Center and Key Laboratory of Biotherapy of Zhejiang Province, Hangzhou, China; ^4^ Affiliated Cixi Hospital, Wenzhou Medical University, Ningbo, China; ^5^ Breast Surgical Department, Shaoxing Maternity and Child Health Care Hospital, Shaoxing, China; ^6^ The First Affiliated Hospital, Zhejiang University School of Medicine, Hangzhou, China

**Keywords:** breast cancer, STAT5A, ABCB1, pimozide, doxorubicin resistance

## Abstract

Chemoresistance is a daunting challenge to the prognosis of patients with breast cancer. Signal transducer and activator of transcription (STAT) 5a plays vital roles in the development of various cancers, but its function in breast cancer is controversial, and its role in chemoresistance in breast cancer remains unexplored. Here we identified STAT5a as a chemoresistance inducer that regulates the expression of ABCB1 in breast cancer and can be targeted by pimozide, an FDA-approved psychotropic drug. First, we found that STAT5a and ABCB1 were expressed at higher levels in doxorubicin-resistant cell lines and chemoresistant patients, and their expression was positively correlated. Then, we confirmed the essential roles of STAT5a and ABCB1 in doxorubicin resistance in breast cancer cells and the regulation of ABCB1 transcription by STAT5a. Subsequently, the efficacy of pimozide in inhibiting STAT5a and sensitizing doxorubicin-resistant breast cancer cells was tested. Finally, we verified the role of STAT5a in doxorubicin resistance in breast cancer and the efficacy of pimozide in reversing this resistance *in vivo*. Our study demonstrated the vital role of STAT5a in doxorubicin resistance in breast cancer. Targeting STAT5a might be a promising strategy for treating doxorubicin-resistant breast cancer. Moreover, repurposing pimozide for doxorubicin resensitization is attractive due to the safety profile of pimozide.

## Introduction

Breast cancer is the most common malignant tumor in women. Every year, 1.7 million people are diagnosed worldwide, and approximately half a million people die from this disease (1). Chemoresistance is a main cause of breast cancer-related death, as it results in recurrence and metastasis. Thus, overcoming this issue is critical to improving the prognosis of patients with breast cancer.

Signal transducer and activator of transcription (STAT) 5a belongs to the STAT family, which consists of seven members (STAT1, STAT2, STAT3, STAT4, STAT5a, STAT5b and STAT6) and participates in essential biological behaviors in cells. Similar to other STATs, STAT5 consists of a helical N-terminal domain (ND), a coiled-coil (CC) domain, a DNA-binding domain (DBD), a helical linker (LK), a Src homology 2 (SH2) domain, and a transactivation domain (TAD) located in the C-terminal region (2). STAT5 is mostly present in the cytoplasm in an inactivated state (3). Upon stimulation by a spectrum of cytokines, STAT5 molecules are recruited to the JAK/receptor complex and phosphorylated at tyrosine (Tyr) 694 (STAT5a) or Tyr 699 (STAT5b). Subsequently, STAT5 translocates to the nucleus in the form of dimers and/or tetramers to function as a transcription factor. Stat5a and stat5b derived from distinct but chromosome-linked genes that map to chromosome 17 (bands q11-1 to q22) (4). These two proteins share 94% identity in their amino acid sequences, with the greatest difference in the C-terminal phosphotyrosyl tail and transactivation domain (5). Although STAT5a and STAT5b have some common target genes, they exert nonredundant functions, resulting in unique target gene activation patterns (6, 7). While STAT5a is mainly present in mammary tissue, STAT5b expression is more enriched in muscle and the liver (8). STAT5a plays a vital role in the promotion of cancers, including lung cancer (9), prostate cancer (10), and gastric cancer (11); is involved in chemoresistance in esophageal cancer by negatively regulating miR-29c (12); and is overexpressed in gemcitabine-resistant pancreatic cancer cell lines (13). STAT5 is also activated and localized to the nucleus in a high proportion of breast cancers (14) and promotes cancer progression (15). DNA-damaging agents such as doxorubicin are reported to induce STAT5a expression in breast cancer (16); however, the exact role of STAT5a in chemoresistance in breast cancer remains unknown.

ABCB1 is one of 49 putative members of the superfamily of human adenosine triphosphate (ATP)-binding cassette (ABC) transporters within subfamily B (MDR/TAP) (17). This membrane transporter is known to promote chemoresistance by exporting antitumor drugs out of cancer cells in various cancers, including breast cancer (18–21).

Here, we identified STAT5a as a key promoter of doxorubicin (DOX) resistance in breast cancer *via* upregulation of the expression of ABCB1. An inhibitor of STAT5, pimozide, which is an FDA-approved drug for treatment of psychotropic diseases, significantly sensitized breast cancer cells to DOX both *in vitro* and *in vivo*.

## Materials & Methods

### Patient Specimens

Breast cancer specimens were obtained from patients (n = 67) with breast cancer at the Department of Surgical Oncology, Sir Run Run Shaw Hospital. The patient population included people with the luminal A (n=10), luminal B (n=22), HER2-positive (n=15) or triple-negative (n=20) subtype, and the patients received doxorubicin-containing neoadjuvant chemotherapy including doxorubicin plus cyclophosphamide (AC), AC-paclitaxel (T) or AC-T plus Herceptin (H). No residual invasive carcinoma in the breast or lymph nodes (noninvasive breast residuals were allowed) assessed by surgical pathological evaluation was defined as a pathologic complete response (pCR). Patients with pCR were defined as chemosensitive, while those with non-pCR were defined as chemoresistant.

### Cell Lines and Regents

The MCF7 cell line was purchased from Cell Bank of the Chinese Academy of Sciences (Shanghai, China) where they were characterized by STR analysis and detection of isozyme, mycoplasma and cell vitality. The cells were maintained in Eagle’s Minimum Essential Medium supplemented with 0.01 mg/ml insulin. DOX-resistant MCF7 cells (MCF7/DOX) were established by DOX (D1515, Sigma-Aldrich, St. Louis, MO, USA) challenge at a starting concentration of 1 ng/ml. The concentration of DOX was gradually increased to 1 μg/ml. Cells were cultured in medium containing 10% FBS in a humidified incubator at 37°C.

### Western Blotting

Protein samples were subjected to sodium dodecyl sulfate-polyacrylamide gel electrophoresis (Bio-Rad, Hercules, CA, USA) and transferred to a polyvinylidene difluoride membrane (Millipore, Billerica, MA, USA) that was blocked in 0.1% Tween-20 in Tris-buffered saline (TBS) containing 5% skim milk (BD Biosciences, Chicago, IL, USA) for 1 h at room temperature and incubated overnight with primary antibodies against STAT5a (ab32043, Abcam, Cambridge, MA, USA), STAT5b (ab178941; Abcam), p-STAT5 (Tyr694) (ab32364, Abcam), cleaved PARP (9541, Cell Signaling Technology, CST, Danvers, MA, USA), cleaved caspase 7 (9491, CST), cleaved caspase 3 (9664, CST), ABCB1 (13978, CST) and β-actin (sc-477748, Santa Cruz, CA, USA). After three 5-min washes with 0.1% Tween-20 in TBS, the membrane was incubated with a diluted horseradish peroxidase (HRP)-conjugated secondary antibody (1:2000, CST). After three 5-min washes with 0.1% Tween-20 in TBS, the membrane was treated with a Pico ECL kit (FDbio, Hangzhou, Zhejiang, China) and imaged with an Amersham Imager 600 (GE Healthcare, Piscataway, NJ, USA).

### RNA Isolation and Quantitative Real-Time PCR

Total RNA was extracted using TRIzol Reagent (Invitrogen, Carlsbad, CA, USA), and the isolated RNA (1 μg) was reverse transcribed with the HiFiScript cDNA Synthesis Kit (CW2569M, CWBIO, Beijing, China). Quantitative real-time PCR was performed using Ultra SYBR Mixture (CW0957H, CWBIO). Glyceraldehyde 3-phosphate dehydrogenase (GAPDH) was used as the reference gene. The following primers were used:

STAT5a: 5′-ATGCTGTTGCCCACGTTTC-3′ (sense),
5′-TGTCCACCCACCATATCCTAGAC-3′ (anti-sense);
ABCB1: 5′-CGAGGTCGGAATGGATCTTGA-3′ (sense),
5′-CCAAAGTTCCCACCACCATATAC-3′ (anti-sense); and
GAPDH: 5′-TGACTTCAACAGCGACACCCA-3′ (sense),
5′-CACCCTGTTGCTGTAGCCAAA-3′ (anti-sense).

Results were calculated using the 2^-△△Ct method.

### Transfection of Plasmids and siRNAs and Infection With a Lentivirus

STAT5a- or ABCB1-overexpressing or negative control vectors were designed and synthesized commercially by GeneChem (Shanghai, China). Short interfering RNAs (siRNAs) targeting STAT5a or ABCB1 (si-STAT5a and si-ABCB1) and a scrambled control siRNA were designed and synthesized commercially by RiboBio (Guangzhou, China). The target sequences of the 3 STAT5a-specific siRNAs were as follows:

sequence 1, 5’-TGATGGAGGTGTTGAAGAA-3’;
sequence 2, 5’-GCAATGAGCTTGTGTTCCA-3’;
sequence 3, 5’-GAGAATTCGACCTGGATGA-3’.

The target sequences of the 3 ABCB1-specific siRNAs were as follows:

sequence 1, 5’-CACTGTTACTCTTAGCAAT-3’;
sequence 2, 5’-GAGCTTAACACCCGACTTA-3’;
sequence 3, 5’-GTGATAGCTCATCGTTTGT-3’.

Transfection of the plasmids and siRNAs into cells was conducted using Lipofectamine 3000 (Invitrogen) transfection reagents following the manufacturer’s instructions. Cells were harvested for total RNA and protein extraction 48 h after transfection and processed for functional assays.

To construct a STAT5a-knockdown MCF7/DOX cell line (MCF7/DOX sh-STAT5a) and corresponding negative control cell line (MCF7/DOX sh-NC), cells were seeded in 6-well plates and incubated for 24 h with lentiviruses (OOBIO, Shanghai, China) carrying shRNA sequences targeting STAT5a (NM_001288718.2, 5’-TGATGGAGGTGTTGAAGAA-3’) or a scrambled sequence (5’-CCTAAGGTTAAGTCGCCCTCG-3’). Seventy-two hours after renewal of the medium, 1 μg/ml puromycin was applied to kill uninfected cells.

### Immunohistochemical (IHC) Staining 

Breast tumor samples were collected from breast cancer patients at Sir Run Run Shaw Hospital. For immunohistochemical analysis, slides were heated in a pressure cooker containing 10 mM sodium citrate (pH 6.5) for 10 min. Endogenous peroxidases were deactivated by treatment with 3% H_2_O_2_ for 5 min, and the slides were blocked with 10% normal goat serum for 30 min at room temperature and probed with primary antibodies against STAT5a (sc-271542X, Santa Cruz), p-STAT5 (Tyr694) (ab32364, Abcam) or ABCB1 (13978, CST) for 1 h at room temperature. The slides were incubated with poly-HRP secondary antibodies for 1 h in the dark at room temperature, and immunodetection was performed using a 3,3′-diaminobenzidine substrate kit. The sections were counterstained with hematoxylin to visualize nuclei. Staining intensity was scored by blinded observers according to intensity and percentage of positive cells. The staining intensity was ranged in four grades: 0 (no staining), 1 (weak staining), 2 (intermediate staining), or 3 (strong staining). The product (percentage of positive cells and respective intensity scores) was used as the final staining score (ranging from 0 to 300).

### DOX Efflux Experiment

To assess the accumulation of DOX in cells, pretreated MCF7 or MCF7/DOX cells were treated with the indicated concentration of DOX for 24 h and then imaged under a microscope (ZEISS, Jena, Germany). The mean and total fluorescence intensities were analyzed with ImageJ software (National Institutes of Health, Bethesda, MD, USA).

### Chemotherapy Sensitivity Assay

Cells were seeded in 96-well plates at a density of 5×10^3^ cells per well in the appropriate medium. The cells were then treated with DOX (at concentrations of 0, 0.01, 0.02, 0.05, 0.1, 0.2, 0.5, 1, and 2 μg/ml) for 24 h. After the incubation, viability was assessed by a CCK8 (APExBio, Houston, TX, USA) assay according to the supplier’s instructions. The absorbance of each well at 450 nm was measured, and survival rate curves were plotted.

### Flow Cytometry

To determine the proportion of apoptotic cells, pretreated cells were collected, washed twice with PBS, and double stained with fluorescein isothiocyanate (FITC)-Annexin V and propidium iodide (PI) (556547, BD Biosciences) according to the supplier’s instructions. The apoptosis rate was measured by flow cytometry on a FACScan (BD Bioscience).

### Colony Formation Assay

To evaluate colony-forming capacity, 2000 pretreated cells per well were seeded in 6-well plates in medium containing an appropriate concentration of DOX, and the medium was replaced as necessary. After 14 days, the cells were washed with PBS 3 times, fixed in 4% paraformaldehyde (Solarbio, Beijing, China) and stained with 0.1% crystal violet (Solarbio). Colonies were counted, and the results were analyzed.

### Luciferase Reporter Assay

A full-length ABCB1 promoter vector was constructed by and purchased from GeneCopoeia (Hangzhou, China). The indicated cells were seeded in 6-well plates and transfected with an ABCB1 reporter plasmid and a STAT5a-overexpressing vector, a negative control vector, STAT5a-specific siRNA, or control siRNA. Forty-eight hours after transfection, the activities of Gaussia luciferase and secreted alkaline phosphatase were assessed using the Secrete-Pair Gaussia Luciferase Assay Kit (LF031, GeneCopoeia) following the manufacturer’s instructions and a 20/20 luminometer (Promega, Madison, WI, USA).

### Chromatin Immunoprecipitation (ChIP) Assay

A ChIP assay was performed using the SimpleChIP^®^ Enzymatic Chromatin IP Kit (Magnetic Beads) (9003, CST) as described previously (22). Chromatin was used for immunoprecipitation with an anti-STAT5a, an anti-histone 3, or a normal rabbit IgG antibody. ChIP-enriched DNA was assessed using real-time PCR, and the primer sets for the ABCB1 promoter were designed as follows:

primer 1, 5′-GCGGTCAGGGAGGTTTCACATCAC-3′ (forward),
5′-CCCATGCATCCGTTTATAGGCTCT-3′ (reverse);
primer 2, 5′-GCTCTTCTACACCTCTTTAGGGT-3′ (forward),
5′-GTAACAGTTGCAACAAAAGCTGG-3′ (reverse);
primer 3, 5′-CTAATTATTTTTTAGCCAGTGGATAAAGAG-3′ (forward),
5′-GCCAGAATAGGCAGAATGAAGATTAGAATC-3′ (reverse); and
primer 4, 5′-CTACTTTATTCAGATATTCTCCAGATTCC-3′ (forward),
5′-CCTTACCTTTTATCTGGTTGCTTCCTG-3′ (reverse).

### Tumor Xenograft Assay

To prove the role of STAT5a in DOX resistance *in vivo*, mice were randomly divided into 2 groups (n=7) and subcutaneously injected with 2 × 10^6^ sh-NC MCF7/DOX or sh-STAT5a MCF7/DOX cells, which were resuspended in 0.1 ml PBS containing 50% Matrigel (Corning, Kennebunk, ME, USA). After one week, the mice were treated with DOX (4 mg/kg, every 3 days, i.p.). To determine the effect of pimozide on STAT5 inhibition and sensitization to DOX, mice were subcutaneously injected with 2 × 10^6^ MCF7/DOX cells as previously described and randomly divided into 4 groups (n=5). After one week, the mice in each group were treated with PBS, pimozide (25 mg/kg, daily, i.p.), DOX (4 mg/kg, every 3 days, i.p.) or both pimozide (25 mg/kg, daily, i.p.) and DOX (4 mg/kg, every 3 days, i.p.). The animals were maintained in laminar flow cabinets under specific pathogen-free conditions. Tumor volumes and mouse body weights were recorded every 3 days. After 21 days, tumor tissue was collected, measured, weighed, fixed in 10% formalin, embedded in paraffin, and processed for hematoxylin and eosin (H&E) and IHC staining.

### Statistical Analysis

Quantitative data from at least three independent experiments are expressed as the mean ± SD. All experimental values were evaluated using GraphPad Prism 9.0.0 (GraphPad Software, Inc., La Jolla, CA, USA). An unpaired t-test was used for statistical analysis of two experimental groups. The relationships between STAT5a or ABCB1 expression and the pCR rate were tested by Fisher’s exact test. Gene expression data obtained from GEO (accession code: GSE87455) were analyzed with GEO2R (23). Patients were divided into STAT5a-low and STAT5a-high groups using the median expression of STAT5a as the cutoff. In all cases, p < 0.05 was considered statistically significant.

## Results

### STAT5a Is Involved in Chemotherapy Resistance in Breast Cancer

**Figure 1:**
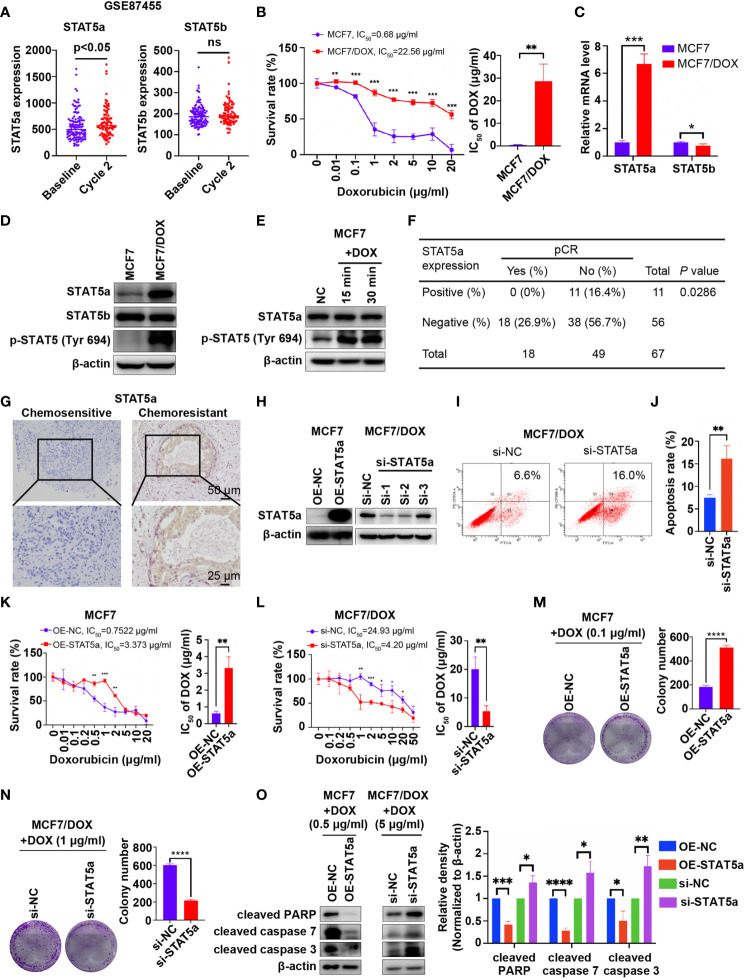
STAT5a is involved in chemoresistance in breast cancer. **(A)** Expression of STAT5a and STAT5b in breast cancer samples collected pre- and postchemotherapy in the dataset GSE87455. **(B)** Survival rates of MCF7 and MCF7/DOX cells after treatment with DOX for 48 h determined by a CCK8 assay. **(C)** mRNA levels of STAT5a and STAT5b in MCF7 and MCF7/DOX cells assessed *via* qPCR. **(D)** Protein levels of STAT5a, p-STAT5a (Tyr694) and STAT5b in MCF7 and MCF7/DOX cells determined by Western blotting. **(E)** Western blotting was performed to examine the expression of STAT5a and p-STAT5 (Tyr694) in MCF7 cells upon treatment with DOX. **(F)** Correlation between the pCR rate and STAT5a expression in breast cancer samples obtained from 67 patients. **(G)** Representative images of IHC staining for STAT5a in chemoresistant and chemosensitive breast cancer samples. **(H)** Efficiency of vector transfection for overexpression of STAT5a in MCF7 cells and siRNA transfection for knockdown of STAT5a in MCF7/DOX cells determined by Western blotting. **(I, J)** Flow cytometry was performed to assess apoptosis in MCF7/DOX cells after knocking down STAT5a or control treatment **(I)**. Bar graphs showing the percentage of apoptotic cells **(J)**. **(K)** Survival rate and IC_50_ of MCF7 cells transfected with an empty vector or a STAT5a vector after treatment with DOX for 48 h determined by a CCK8 assay. **(L)** Survival rate and IC_50_ of MCF7/DOX cells transfected with scramble siRNA or STAT5a-targeting siRNA after treatment with DOX for 48 h determined by a CCK8 assay. **(M, N)** Representative images and quantification of colonies formed by MCF7 cells transfected with the empty vector or STAT5a vector **(M)** and MCF7/DOX cells transfected with scramble siRNA or STAT5a-targeting siRNA **(N)** in medium containing the indicated concentration of DOX. **(O)** The expression levels of apoptosis markers in MCF7 cells transfected with the empty vector or STAT5a vector and MCF7/DOX cells transfected with scramble siRNA or STAT5a-targeting siRNA under treatment with the indicated concentration of DOX determined by Western blotting. ns, p > 0.05; *p < 0.05; **p < 0.01; ***p < 0.001; ****p < 0.0001.

Data mining based on GSE87455 was conducted to explore whether STAT5a is involved in chemoresistance in breast cancer (23). STAT5a expression was potently upregulated in the postchemotherapy group, while STAT5b showed no significant difference, implying a role for STAT5a but not STAT5b in chemoresistance (**Figure 1A**). Then, we verified the data mining results *in vitro* and *in vivo*. First, we compared the IC_50_ values of wild-type MCF7 and our DOX-resistant MCF7 (MCF7/DOX) cells, which were maintained in medium containing 1 μg/ml DOX for more than 6 months, by a CCK8 assay. A 32-fold increase in the IC_50_ of DOX was observed in MCF7/DOX cells compared with MCF7 cells (**Figure 1B**). The mRNA and protein expression of STAT5a but not that of STAT5b was upregulated in the MCF7/DOX cell line, and the level of STAT5a phosphorylation at Tyr694 was also higher in MCF7/DOX cells (**Figures 1C, D**). Additionally, we observed activation of STAT5a upon stimulation of MCF7 cells with DOX, implying a role for STAT5a in the response to chemotherapy stress (**Figure 1E**). Given that STAT5a but not STAT5b was upregulated and activated in chemoresistant patients and cell lines, we focused on STAT5a in the rest of the study. We applied our clinical data to verify the data mining result. IHC was applied to assess the level of STAT5a in breast tumor specimens collected from patients before neoadjuvant treatment. Patients diagnosed as pCR after operation were defined chemo-sensitive, while others chemoresistant. Statistical analysis suggested that patients with STAT5a-positive breast cancer had a lower pCR rate than STAT5a-negative patients (**Figure 1F**). Representative IHC results of STAT5a expression in chemosensitive and chemoresistant patients were shown in **Figure 1G**.

To investigate whether STAT5a can regulate the sensitivity of breast cancer cells to DOX, we knocked down and overexpressed STAT5a *via* siRNA and a plasmid in MCF7/DOX and MCF7 cells, respectively (**Figure 1H**). Si-1 siRNA was selected for subsequent experiments due to its knockdown efficiency. An increased apoptosis rate was observed in STAT5a-knockdown MCF7/DOX cells (**Figures 1I, J**), suggesting a role for STAT5a in cell survival. When STAT5a was overexpressed, MCF7 cells became more resistant to DOX, with the IC_50_ increasing from 0.7522 μg/ml to 3.373 μg/ml (**Figure 1K**). STAT5a knockdown also sensitized MCF7/DOX cells to DOX, decreasing the IC_50_ from 24.93 μg/ml to 4.2 μg/ml, as assessed by CCK8 assays (**Figure 1L**). The capacity to form colonies in the presence of DOX was enhanced by STAT5a upregulation in MCF7 cells and attenuated by STAT5a knockdown in MCF7/DOX cells (**Figures 1M, N**). Treatment with DOX induced less apoptosis in STAT5a-overexpressing MCF7 cells and more apoptosis in STAT5a-knockdown MCF7/DOX cells, as indicated by analysis of biomarkers of apoptosis, such as cleaved PARP and cleaved caspase 3/7 (**Figure 1O**). These data suggested that STAT5a could confer DOX resistance in breast cancer.

### ABCB1 Is Essential to Chemoresistance in Breast Cancer

**Figure 2:**
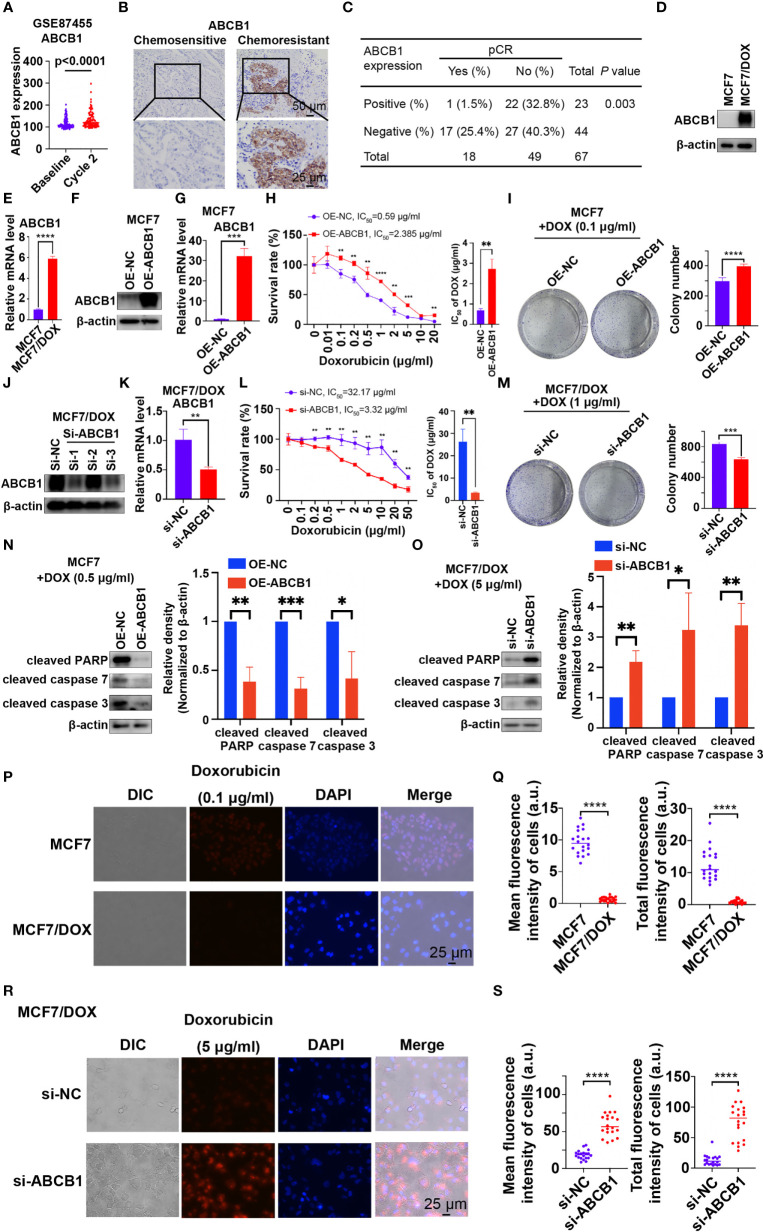
ABCB1 is essential to chemoresistance in breast cancer. **(A)** Expression of ABCB1 in breast cancer samples collected pre- and postchemotherapy in the dataset GSE87455. **(B)** Representative images of IHC staining for ABCB1 in chemoresistant and chemosensitive breast cancer samples. **(C)** Correlation between the pCR rate and ABCB1 expression in breast cancer samples obtained from 67 patients. **(D, E)** Protein **(D)** and mRNA **(E)** levels of ABCB1 in MCF7 and MCF7/DOX cells determined by Western blotting and qPCR. **(F, G)** Protein **(F)** and mRNA **(G)** levels of ABCB1 in MCF7 cells transfected with an empty vector or ABCB1 vector determined by Western blotting and qPCR. **(H)** Survival rate and IC_50_ of MCF7 cells transfected with an empty vector or a STAT5a vector after treatment with DOX for 48 h determined by a CCK8 assay. **(I)** Representative images and quantification of colonies formed by MCF7 cells transfected with the empty vector or ABCB1 vector in medium containing DOX. **(J, K)** Protein **(J)** and mRNA **(K)** levels of ABCB1 in MCF7/DOX cells transfected with scramble siRNA or ABCB1-targeting siRNA detected by Western blotting and qPCR. **(L)** Survival rate and IC_50_ of MCF7/DOX cells transfected with scramble siRNA or ABCB1-targeting siRNA after treatment with DOX for 48 h determined by a CCK8 assay. **(M)** Representative images and quantification of colonies formed by MCF7/DOX cells transfected with scramble siRNA or ABCB1-targeting siRNA in medium containing DOX. **(N, O)** Expression of apoptosis markers in MCF7 cells transfected with the empty vector or ABCB1 vector **(N)** and in MCF7/DOX cells transfected with scramble siRNA or ABCB1-targeting siRNA in the presence of DOX **(O)**. **(P, Q)** Representative images **(P)** and quantification **(Q)** of the accumulation of DOX in MCF7 and MCF7/DOX cells after treatment with DOX. **(R, S)** Representative images **(R)** and quantification **(S)** of the accumulation of DOX in MCF7/DOX cells transfected with scramble siRNA or ABCB1-targeting siRNA after DOX treatment. *p < 0.05; **p < 0.01; ***p < 0.001; ****p < 0.0001.

ABCB1, a well-known multidrug resistance protein, plays a vital role in chemoresistance in various types of cancers (18–21). ABCB1 expression was significantly increased in patients who received 2 cycles of chemotherapy (“Cycle 2”) compared to prechemotherapy (“Baseline”) expression in the dataset GSE87455 (**Figure 2A**), suggesting the development of chemoresistance after chemotherapy (23). We also investigated the role of ABCB1 in chemoresistance in our study. First, the level of ABCB1 was significantly higher in tumor tissues from patients with chemoresistant breast cancer than in those from chemosensitive patients (**Figure 2B**). Statistical analysis showed that patients with ABCB1-positive breast cancer had a significantly lower pCR rate than those with ABCB1-negative breast cancer (**Figure 2C**). Furthermore, qPCR and Western blot results showed that the mRNA and protein levels of ABCB1 were significantly higher in the MCF7/DOX cell line than in the MCF7 cell line (**Figures 2D, E**). Then, we overexpressed and knocked down ABCB1 *via* a plasmid and siRNA in MCF7 and MCF7/DOX cells, respectively (**Figures 2F, G, J, K**). Si-1 siRNA was selected for subsequent experiments. When the expression of ABCB1 was upregulated, resistance of MCF7 cell to DOX was increased, with the IC_50_ elevated from 0.59 μg/ml to 2.385 μg/ml (**Figure 2H**), and the colony-forming ability in the presence of DOX was also increased (**Figure 2I**). The expression of biomarkers of apoptosis was significantly reduced in ABCB1-overexpressing MCF7 cells upon treatment with DOX (**Figure 2N**). On the other hand, ABCB1 knockdown in MCF7/DOX cells showed the opposite effects. When ABCB1 was downregulated, MCF7/DOX cells became less resistant to DOX, with the IC_50_ decreasing from 32.17 μg/ml to 3.32 μg/ml (**Figure 2L**), and fewer colonies formed in the presence of DOX (**Figure 2M**). DOX-induced apoptosis was increased in ABCB1-silenced MCF7/DOX cells, as indicated by assessment of biomarkers of apoptosis (**Figure 2O**). Since ABCB1 confers cell chemoresistance by pumping drugs out of cells, we compared the amount of DOX in MCF7 and MCF7/DOX cells and explored whether the expression of ABCB1 alters the amount of DOX in cells. DOX was monitored *via* fluorescence microscopy due to its intrinsic fluorescence (24). As shown in **Figures 2P, Q**, the amount of DOX in MCF7/DOX cells was significantly lower than that in MCF7 cells, implying that MCF7/DOX cells had a more potent ability to pump out drugs. When ABCB1 was knocked down, the amount of DOX in cells was significantly elevated (**Figures 2R, S**), implying an impaired ability to pump out drugs. Thus, ABCB1 is essential to chemoresistance in breast cancer cells by pumping drugs out of cancer cells.

### STAT5a Modulates Chemoresistance in Breast Cancer by Regulating the Transcription of ABCB1

**Figure 3:**
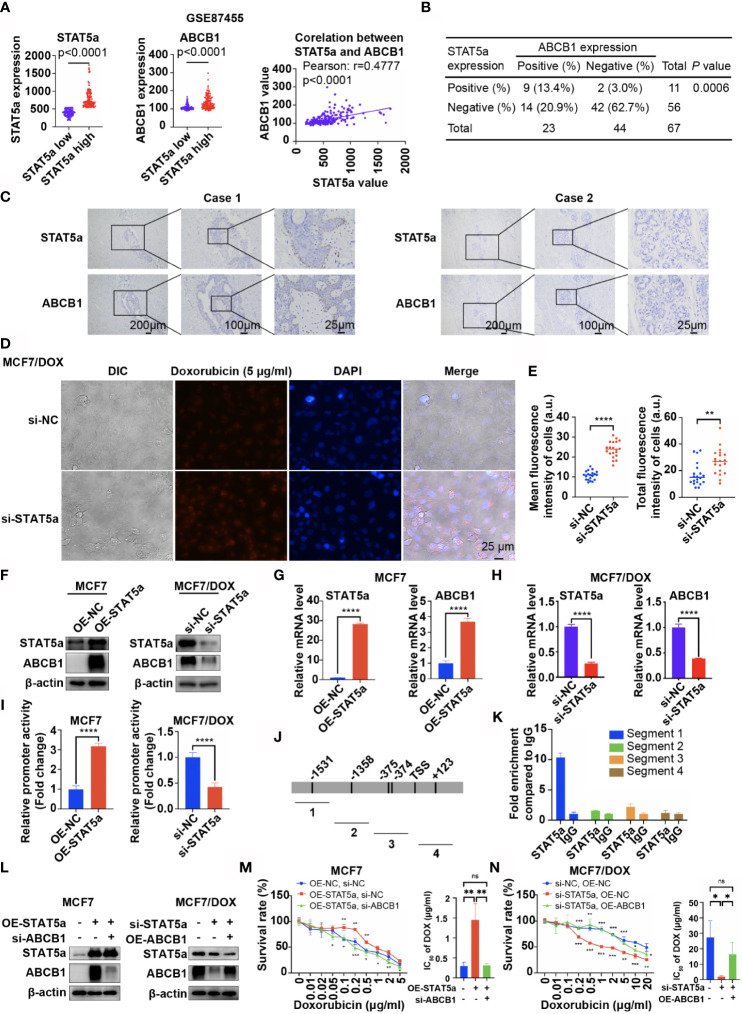
STAT5a modulates chemoresistance in breast cancer by regulating the transcription of ABCB1. **(A)** Data from GSE87455 showed that the expression of ABCB1 was higher in the STAT5a-high group than in the STAT5a-low group in breast cancer, and the expression of STAT5a and ABCB1 was positively correlated. **(B)** Correlation between STAT5a expression and ABCB1 expression in breast cancer samples obtained from 67 patients. **(C)** Representative images of STAT5a and ABCB1 expression levels in breast cancer samples. **(D, E)** Representative images **(D)** and quantification **(E)** of the accumulation of DOX in MCF7/DOX cells transfected with scramble siRNA or STAT5a-targeting siRNA after treatment with DOX. **(F)** Expression levels of STAT5a and ABCB1 in MCF7 cells transfected with an empty vector or a STAT5a vector determined by Western blotting and MCF7/DOX cells transfected with scramble siRNA or STAT5a-targeting siRNA. **(G, H)** mRNA levels of STAT5a and ABCB1 in MCF7/DOX cells transfected with scramble siRNA or STAT5a-targeting siRNA **(G)** and in MCF7/DOX cells transfected with scramble siRNA or STAT5a-targeting siRNA **(H)** determined by qPCR. **(I)** Relative promoter activity in MCF7 cells transfected with the empty vector or STAT5a vector and in MCF7/DOX cells transfected with scramble siRNA or STAT5a-targeting siRNA. **(J)** Four pairs of primers were designed to detect sequences covering five predicted binding sites for STAT5a in the ABCB1 promoter region. **(K)** Binding between STAT5a and the ABCB1 promoter region in sequence 1 was determined by ChIP. **(L)** Levels of STAT5a and ABCB1 in MCF7 cells transfected with the indicated vector or siRNA and in MCF7/DOX cells transfected with the indicated targeting siRNA or vectors. **(M, N)** Survival rates and IC_50_ of MCF7 **(M)** and MCF7/DOX **(N)** cells transfected with the indicated vector or siRNA after treatment with DOX for 48 h determined by a CCK8 assay. ns, p > 0.05; *p < 0.05; **p < 0.01; ***p < 0.001; ****p < 0.0001.

Data from GSE87455 also showed that the expression level of ABCB1 was significantly higher in the STAT5a-high group (STAT5a level higher than the median) than in the STAT5a-low group (STAT5a level lower than the median) and positively correlated with the expression level of STAT5a (**Figure 3A**). IHC results for our clinical specimens also suggested a significant relationship between STAT5a and ABCB1, with a p value of 0.0006 (**Figure 3B**). Breast cancer specimens with high STAT5a expression showed high ABCB1 expression levels (**Figure 3C**). When STAT5a was knocked down, the ability of MCF7/DOX cells to pump out DOX was attenuated (**Figures 3D, E**). To explore the relationship between STAT5a and ABCB1, we tested whether STAT5a could regulate the expression of ABCB1. The results showed that overexpressing STAT5a in MCF7 cells significantly increased the expression of ABCB1 at both the protein and mRNA levels, and STAT5a knockdown in MCF7/DOX cells had the opposite effect (**Figures 3F–H**).

To further explore how STAT5a modulates ABCB1 expression in breast cancer cells, a 2012-bp fragment of DNA containing ABCB1 sequences from −1686 to 326 relative to the transcription initiation site was subcloned into the Pezx-PG04.1 vector. We cotransfected the ABCB1 promoter vector with a STAT5a expression vector into MCF7 cells or with STAT5a-targeting siRNAs into MCF7/DOX cells. The results in **Figure 3I** indicate that STAT5a overexpression increased ABCB1 promoter activity, while knocking down STAT5a expression attenuated ABCB1 promoter activity. To verify the interactions between STAT5a and five potential STAT5a-binding sites in the ABCB1 promoter region (**Figure 3J**), a chromatin immunoprecipitation (ChIP) assay was performed with MCF7/DOX cells and an anti-STAT5a antibody (**Figure 3K**). The results suggested that STAT5a could promote the transcription of ABCB1 by binding to its promoter region.

Subsequently, a rescue experiment was performed to confirm whether STAT5a modulates chemoresistance in breast cancer cells by regulating ABCB1. As shown in **Figures 3L, M**, upregulation of STAT5a increased resistance to DOX in MCF7 cells, and additional downregulation of ABCB1 significantly suppressed resistance. Similar results are shown in **Figures 3L, N**, where knockdown of STAT5a attenuated the resistance of MCF7/DOX cells to DOX, while additional overexpression of ABCB1 significantly recovered DOX resistance. Taken together, these results suggested that STAT5a conferred DOX resistance to breast cancer cells by regulating the transcription of ABCB1.

### STAT5 Inhibitor Pimozide Sensitizes Breast Cancer Cells to DOX

**Figure 4:**
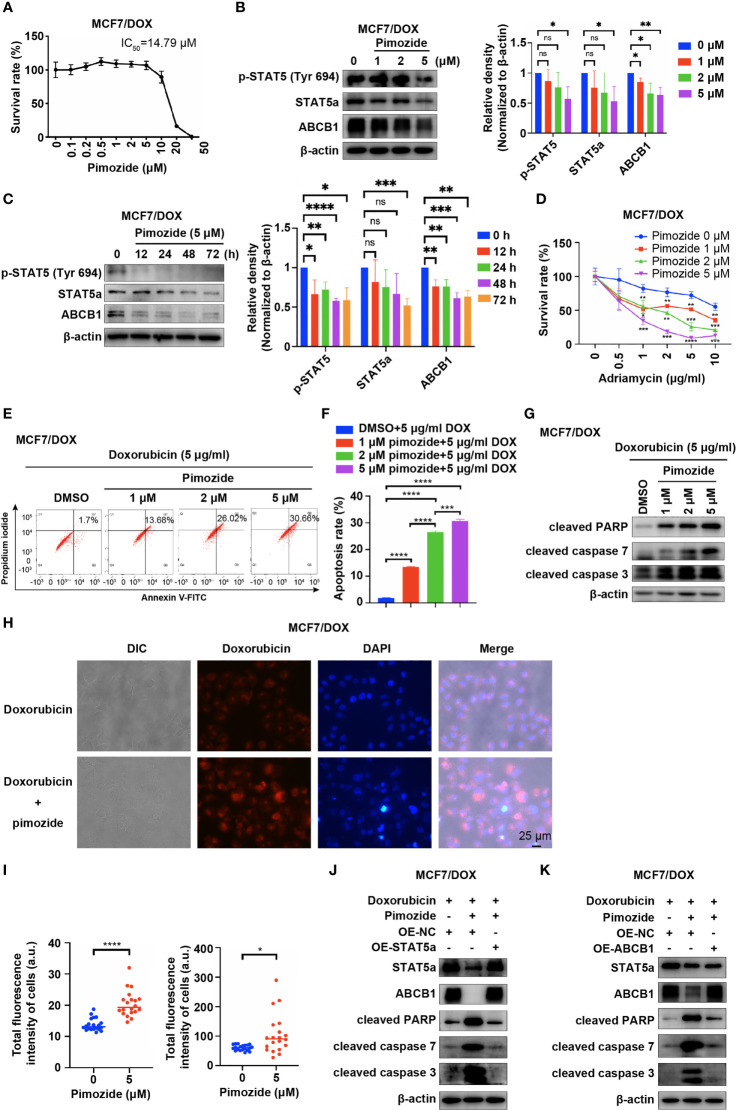
Pimozide sensitizes DOX-resistant cells to DOX by suppressing STAT5a. **(A)** Survival rate of MCF/DOX cells after treatment with pimozide for 48 h, with the IC50 calculated to be 14.79 μM. **(B, C)** Expression of p-STAT5 (Try 694), STAT5a and ABCB1 in MCF7/DOX cells after treatment with 0, 1, 2, or 5 μM DOX for 48 h **(B)** or with 5 μM DOX for 0, 12, 24, 48 or 72 h **(C)**. **(D)** Survival rate of MCF/DOX cells after treatment with a combination of 0, 1, 2 or 5 μM pimozide and the indicated concentration of DOX for 48 h. **(E, F)** Apoptosis rate of MCF7/DOX cells after the indicated treatment assessed by flow cytometry **(E)**; bar graphs showing the percentage of apoptotic cells **(F)**. **(G)** Expression of apoptosis markers in MCF7/DOX cells given the indicated treatments determined by Western blotting. **(H, I)** Accumulation of DOX in MCF7/DOX cells after treatment with DOX or a combination of DOX and pimozide **(H)** and quantification **(I)**. **(J, K)** Western blotting was performed to determine the expression of STAT5a, ABCB1, cleaved PARP, cleaved caspase 7 and cleaved caspase 3 in MCF7/DOX cells treated with DOX and transfected with the indicated vectors. ns, p > 0.05; *p < 0.05; **p < 0.01; ***p < 0.001; ****p < 0.0001.

Pimozide, an FDA-approved drug for treatment of psychotropic diseases, has been applied as a potent inhibitor of STAT5 in numerous studies (25–27). We also applied pimozide in our study to inhibit the activation of STAT5a and tested whether this treatment sensitizes MCF7/DOX cells to DOX. First, we determined the IC_50_ of pimozide in MCF7/DOX cells *via* a CCK8 assay. The results showed that the IC_50_ of pimozide in MCF7/DOX cells was 14.79 μM and that pimozide exhibited negligible cytotoxicity below a concentration of 5 μM (**Figure 4A**). WB results showed that pimozide also inhibited STAT5a in MCF7/DOX cells by suppressing the phosphorylation of STAT5a at Tyr694 in a time- and concentration (1 μM, 2 μM and 5 μM)-dependent manner and that the expression of STAT5a and ABCB1 was also repressed by the administration of pimozide (**Figures 4B, C**). Subsequently, we compared the anticancer effects of DOX and a combination of DOX and pimozide. The results showed that pimozide could significantly sensitize MCF7/DOX cells to DOX in a dose-dependent manner (**Figure 4D**). Flow cytometry experiments indicated that the addition of pimozide significantly increased the apoptosis rate of MCF7/DOX cells treated with DOX in a dose-dependent manner (**Figures 4E, F**). The addition of pimozide also increased the levels of apoptosis biomarkers, such as cleaved PARP and cleaved caspase 3/7, as indicated by Western blot analysis (**Figure 4G**). We also analyzed the influence of pimozide on the ability of breast cancer cells to pump out drugs. The results showed that when pimozide was administered, the accumulation of DOX within cells was remarkably increased in MCF7/DOX cells (**Figures 4H, I**). To confirm that pimozide sensitized breast cancer cells by suppressing the STAT5a/ABCB1 pathway, a rescue experiment was conducted. As shown in **Figures 4J, K**, the addition of pimozide to DOX treatment significantly elevated the expression of cleaved PARP and cleaved caspase 3/7 in MCF7/DOX cells, whereas overexpression of STAT5a or ABCB1 restored the expression to low levels. These data suggested that the STAT5 inhibitor pimozide attenuated the ability of cells to pump out drugs and sensitized DOX-resistant breast cancer cells to DOX by suppressing the STAT5a/ABCB1 pathway.

### STAT5a Knockdown Sensitizes Breast Cancer Cells to DOX *In Vivo*

**Figure 5:**
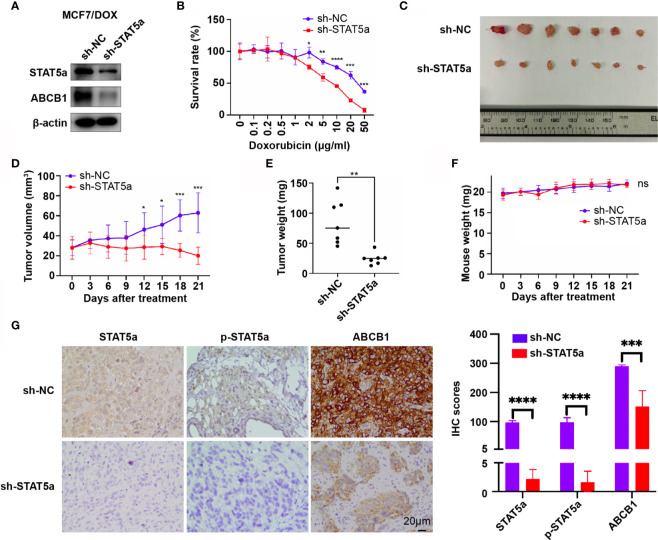
STAT5a knockdown sensitizes breast cancer cells to DOX *in vivo*. **(A)** Expression of STAT5a and ABCB1 in MCF7/DOX sh-NC cells and MCF7/DOX sh-STAT5a cells determined by Western blotting. **(B)** Survival rates of MCF7/DOX sh-NC and MCF7/DOX sh-STAT5a cells after treatment with DOX for 48 h. **(C)** Isolated subcutaneous tumors. **(D)** Tumor growth curves. **(E)** Weights of isolated tumors. **(F)** Nude mouse weight curves. **(G)** Expression of STAT5a, p-STAT5a and ABCB1 in MCF7/DOX sh-NC and MCF7/DOX sh-STAT5a tumors determined by IHC analysis. ns, p > 0.05; *p < 0.05; **p < 0.01; ***p < 0.001; ****p < 0.0001.

To validate our findings *in vivo*, MCF7/DOX sh-NC and MCF7/DOX sh-STAT5a cell lines were constructed, and their expression of STAT5a and ABCB1 and sensitivity to DOX were validated. The expression of STAT5a and ABCB1 was significantly downregulated in MCF7/DOX sh-STAT5a cells (**Figure 5A**), and these cells showed significantly less resistance to DOX than MCF7/DOX sh-NC cells (**Figure 5B**). These two cell lines were used to establish a xenograft tumor model in nude mice (n=7). The results showed that DOX negligibly suppressed the growth of MCF7/DOX sh-NC tumors due to resistance, while MCF7/DOX sh-STAT5a tumors showed a significantly greater response to DOX (**Figures 5C–E** and **Supplementary Figure 1**). There was no difference in mouse body weight between the 2 groups during the treatment period (**Figure 5F**). IHC results showed decreased expression of STAT5a, p-STAT5a and ABCB1 in MCF7/DOX shSTAT5a tumors (**Figure 5G**). These data suggested that knocking down STAT5a decreased the expression of ABCB1 and sensitized chemoresistant breast cancer cells to DOX *in vivo.*

### Pimozide Sensitizes Breast Cancer Cells to DOX *In Vivo*

**Figure 6:**
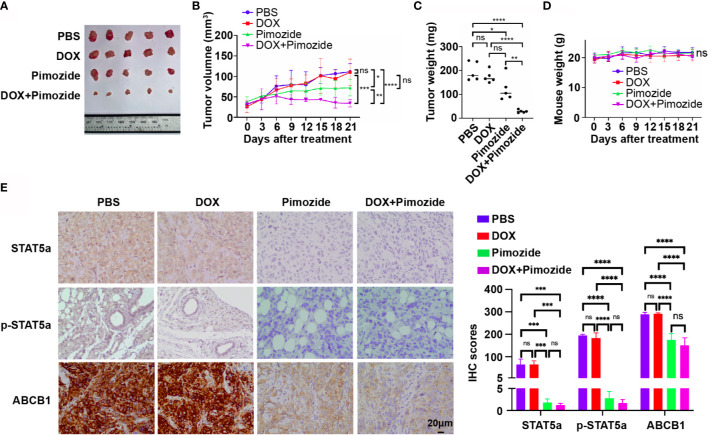
Pimozide sensitizes breast cancer cells to DOX *in vivo*. **(A)** Isolated subcutaneous tumors of each group. **(B)** Growth curves of tumors in each group. **(C)** Weights of isolated tumors in each group. **(D)** Nude mouse weight curves. **(E)** Expression of STAT5a, p-STAT5a and ABCB1 in each group of tumors determined by IHC analysis. ns, p > 0.05; *p < 0.05; **p < 0.01; ***p < 0.001; ****p < 0.0001.

To verify the efficacy of pimozide in the sensitization of breast cancer cells to chemotherapy *in vivo*, an MCF7/DOX xenograft tumor model was established in nude mice. Mice with xenograft tumors were divided into 4 groups (n=5) and received PBS, DOX, pimozide or a combination of DOX and pimozide as described in the methods. As shown in **Figures 6A–C** and **Supplementary Figure 2**, the volume, growth rate, and weight of tumors in the DOX group were not different from those of tumors in the PBS group, while those of tumors in the pimozide group were significantly lower, and those of tumors in the combination group were the lowest. DOX failed to suppress tumor growth due to resistance, while pimozide exhibited both antitumor activity and efficient sensitization to DOX. Mouse body weights were recorded, and no significant differences were observed among the four groups of mice, implying the drug dose was tolerated (**Figure 6D**). The expression of STAT5a, p-STAT5a and ABCB1 detected by IHC was significantly decreased in tumor tissues from the pimozide group and combination group but did not vary between the DOX group and PBS group (**Figure 6E**), suggesting the potent effect of pimozide on suppressing STAT5a and downstream ABCB1. These results demonstrated that a minimally toxic dose of pimozide had an antitumor effect on breast cancer and was able to sensitize breast cancer to DOX *in vitro* and *in vivo*.

## Discussion

Chemoresistance is the leading cause of therapy failure and mortality in breast cancer (28). The drug efflux pump ABCB1 plays a key role in chemoresistance by effluxing various chemotherapeutic agents from tumor cells (21, 28, 29), and its expression is negatively correlated with the prognosis of cancers, including that of breast cancer (30, 31). We verified the function of ABCB1 in chemoresistance in breast cancer cells in our study and proved its regulation by STAT5a.

STAT5a is a member of the STAT family and is highly homologous to STAT5b. STAT5a is mainly present in mammary tissue, while STAT5b is generally expressed in muscle and the liver (8). The role of STAT5a in hematopoietic neoplasms, especially in myeloid cell transformation, is broadly accepted (32), and STAT5a also promotes the development of several other cancers (9–11, 33). However, the role of STAT5a in the development of breast cancer is controversial (34). On the one hand, STAT5a promotes transformation of mammary epithelial cells and survival of breast cancer cells (35, 36); on the other hand, STAT5a is associated with a relatively good prognosis for patient survival since it promotes mammary epithelial cell differentiation (37–40). The results of our study showed that STAT5a was overexpressed and persistently activated in a chemoresistant breast cancer cell line and upregulated ABCB1 expression by promoting its transcription. The interaction of STAT5a and the promoter of ABCB1 was verified by ChIP in our study, and the interaction of STAT5 with a nearby region was also implied by a previous study (41). The roles of STAT5a and ABCB1 in chemoresistance in breast cancer and their regulation were further confirmed by IHC analysis of breast cancer tissues from 67 patients who received DOX-containing neoadjuvant treatment. The pCR rate was used to measure the sensitivity of patients to chemotherapy. We found that STAT5a- or ABCB1-expressing patients exhibited a significantly lower pCR rate, implying the vital roles of STAT5a and ABCB1 in chemoresistance in breast cancer. Moreover, the expression of STAT5a and ABCB1 was positively correlated, which was consistent with our *in vitro* studies.

Pimozide, an FDA-approved psychotropic drug, inactivated STAT5a and downregulated the expression of ABCB1, thus sensitizing MCF7/DOX cells to DOX *in vitro* and *in vivo*, providing a promising strategy for treating patients with chemoresistant breast cancer in the clinic. In fact, pimozide has been found to have antitumor activity mediated by suppressing STAT5 in various types of cancer (25, 42–44). In breast cancer, pimozide has also been proven to promote apoptosis by inhibiting RAN GTPase and AKT and to inhibit epithelial-mesenchymal transition and cell migration (45). Pimozide also acts as a STAT5 inhibitor (46) or STAT3 inhibitor (47) to kill breast cancer cells or sensitize cancer cells to other drugs (48). Additionally, pimozide was found to inhibit ABCB1 in drug-resistant KBV20C oral cancer cells, but the authors did not explain the mechanism (49). Thus, the exact mechanism by which pimozide exerts its anticancer activity remains unclear. In our study, pimozide inhibited STAT5a and ABCB1 in a dose-dependent manner and sensitized breast cancer cells to DOX, which could be rescued by overexpression of STAT5a or ABCB1. The results suggested that the STAT5a/ABCB1 pathway was at least one, if not the main, mechanism by which pimozide functions in drug-resistant breast cancer.

## Conclusions

**Figure 7:**
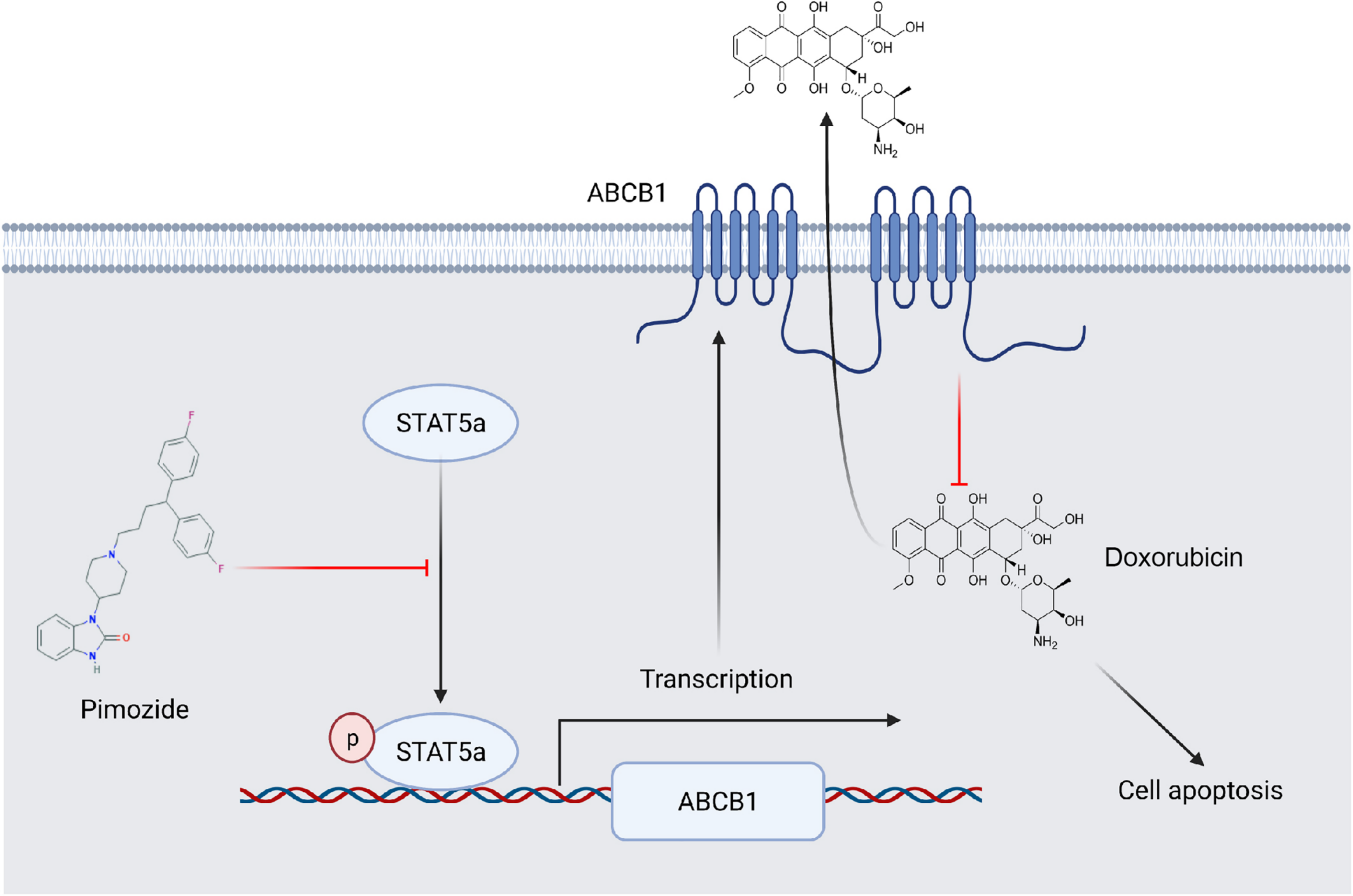
A schematic showing the involvement of STAT5a in DOX resistance in breast cancer. ABCB1 transports DOX out of breast cancer cells to reduce its cellular accumula\tion. STAT5a promotes the transcription of ABCB1 to confer DOX resistance to cells. Pimozide overcomes this resistance by inhibiting the STAT5a/ABCB1 axis.

STAT5a confers breast cancer chemoresistance by upregulating the transcription of ABCB1. Pimozide sensitizes breast cancer cells to DOX by suppressing the activation of STAT5a and downregulating ABCB1 (**Figure 7**). Our study established the vital role of STAT5a in chemoresistance in breast cancer and verified the mechanism. We also identified STAT5a as a therapeutic target for treatment of chemoresistant breast cancer and pimozide as a promising candidate to reduce chemoresistance.

## Data Availability Statement

The original contributions presented in the study are included in the article/**Supplementary Material**. Further inquiries can be directed to the corresponding authors.

## Ethics Statement

The studies involving human participants were reviewed and approved by the Ethics Committee of the Sir Run Run Shaw Hospital at the Zhejiang University School of Medicine. The patients/participants provided their written informed consent to participate in this study. The animal study was reviewed and approved by the Animal Care and Use Committee of Sir Run Run Shaw Hospital, Zhejiang University, Zhejiang, China.

## Author Contributions

LW, JZ, and ZL designed the study, and ZL, CC, LC, DH, and XY performed the experiments. ZL, WZ, and CC contributed to writing the manuscript. YC, JY, YZ, MM, and LX analyzed the data. XZ, SJ, JS, and QinW contributed to manuscript review and revision. MD, SX, QunW, and YJ provided technical support. All authors contributed to the article and approved the submitted version.

## Funding

The work was supported by the National Natural Science Foundation of China (No. 81672729, No. 81972597, No. 81602471, No. 81972453, No. 81672840 and No. 82000212), Zhejiang Provincial Natural Science Foundation of China under Grants (No. LY19H160059, No. LY19H160055, No. LY18H160030 and No. LQ21H160022), Zhejiang Provincial Medical and Health Science and Technology Project (No. 2018ZD028 and No. 2021RC003), Ningbo Natural Science Foundation (No. 2019A610315), and Cixi Agricultural and Social Development Science and Technology Project (No. CN2020006). The work was sponsored by the Zheng Shu Medical Elite Scholarship Fund.

## Conflict of Interest

The authors declare that the research was conducted in the absence of any commercial or financial relationships that could be construed as a potential conflict of interest.

## Supplementary Material

Supplementary Figure 1 Tumors formed in nude mice injected with MCF7/DOX sh-NC cells or MCF7/DOX sh-STAT5a cells and treated with doxorubicin (n=7).

Supplementary Figure 2 Nude mice bearing tumors formed from MCF7/DOX cells were divided into four groups and received the indicated treatment (n=5).

## Abbreviations

STAT, signal transducer and activator of transcription; DOX, doxorubicin; AC, cyclophosphamide; pCR, pathologic complete response.
