# Corrigendum: STAT5a Confers Doxorubicin Resistance to Breast Cancer by Regulating ABCB1

**DOI:** 10.3389/fonc.2022.880458

**Published:** 2022-03-09

**Authors:** Zhaoqing Li, Cong Chen, Lini Chen, Dengdi Hu, Xiqian Yang, Wenying Zhuo, Yongxia Chen, Jingjing Yang, Yulu Zhou, Misha Mao, Xun Zhang, Ling Xu, Siwei Ju, Jun Shen, Qinchuan Wang, Minjun Dong, Shuduo Xie, Qun Wei, Yunlu Jia, Jichun Zhou, Linbo Wang

**Affiliations:** ^1^ Cancer Institute (Key Laboratory of Cancer Prevention and Intervention, China National Ministry of Education), 2nd Affiliated Hospital, School of Medicine, Zhejiang University, Hangzhou, China; ^2^ Sir Run Run Shaw Hospital, Zhejiang University, Hangzhou, China; ^3^ Biomedical Research Center and Key Laboratory of Biotherapy of Zhejiang Province, Hangzhou, China; ^4^ Affiliated Cixi Hospital, Wenzhou Medical University, Ningbo, China; ^5^ Breast Surgical Department, Shaoxing Maternity and Child Health Care Hospital, Shaoxing, China; ^6^ The First Affiliated Hospital, Zhejiang University School of Medicine, Hangzhou, China

**Keywords:** breast cancer, STAT5A, ABCB1, pimozide, doxorubicin resistance

In the original article, there was a mistake in **Figure 1** as published. In **Figure 1F**, the number “42” should be “38”. The corrected **Figure 1** appears below.

**Figure 1:**
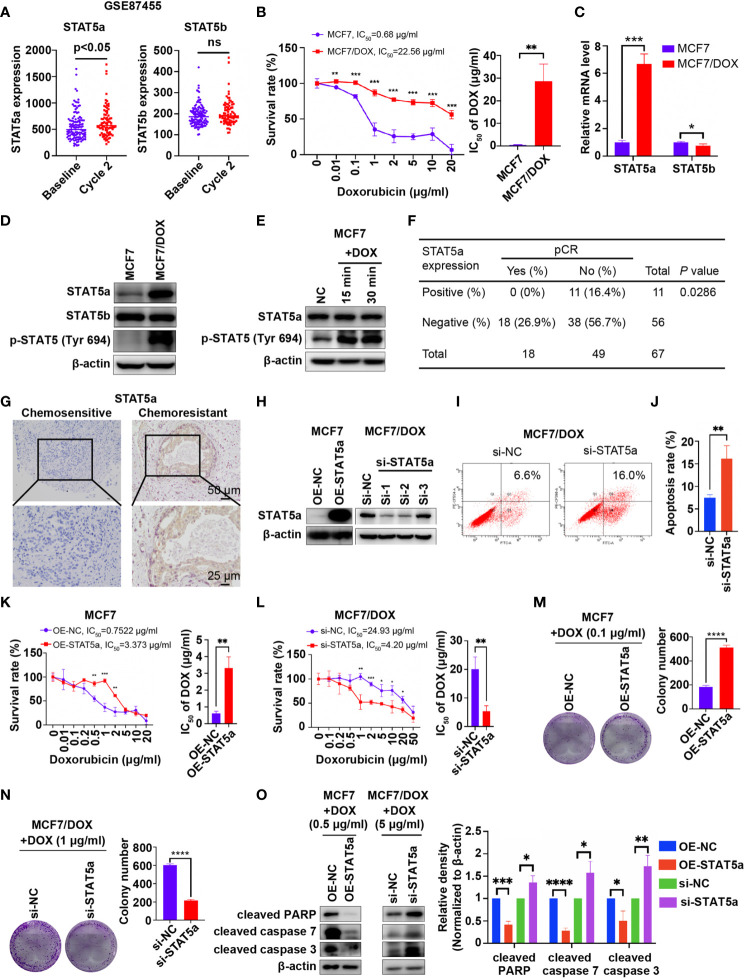
STAT5a is involved in chemoresistance in breast cancer. **(A)** Expression of STAT5a and STAT5b in breast cancer samples collected pre- and postchemotherapy in the dataset GSE87455. **(B)** Survival rates of MCF7 and MCF7/DOX cells after treatment with DOX for 48 h determined by a CCK8 assay. **(C)** mRNA levels of STAT5a and STAT5b in MCF7 and MCF7/DOX cells assessed via qPCR. **(d)** Protein levels of STAT5a, p-STAT5a (Tyr694) and STAT5b in MCF7 and MCF7/DOX cells determined by Western blotting. **(E)** Western blotting was performed to examine the expression of STAT5a and p-STAT5 (Tyr694) in CF7 cells upon treatment with DOX. **(F)** Correlation between the pCR rate and STAT5a expression in breast cancer samples obtained from 67 patients. **(G)** Representative images of IHC staining for STAT5a in chemoresistant and chemosensitive breast cancer samples. **(H)** Efficiency of vector transfection for overexpression of STAT5a in MCF7 cells and siRNA transfection for knockdown of STAT5a in MCF7/DOX cells determined by Western blotting. **(I, J)** Flow cytometry was performed to assess apoptosis in MCF7/DOX cells after knocking down STAT5a or control treatment **(I)**. Bar graphs showing the percentage of apoptotic cells **(J, K)** Survival rate and IC50 of MCF7 cells transfected with an empty vector or a STAT5a vector after treatment with DOX for 48 h determined by a CCK8 assay. **(L)** Survival rate and IC50 of MCF7/DOX cells transfected with scramble siRNA or STAT5a-targeting siRNA after treatment with DOX for 48 h determined by a CCK8 assay. **(M, N)** Representative images and quantification of colonies formed by MCF7 cells transfected with the empty vector or STAT5a vector (M) and MCF7/DOX cells transfected with scramble siRNA or STAT5a-targeting siRNA (N) in medium containing the indicated concentration of DOX. **(O)** The expression levels of apoptosis markers in MCF7 cells transfected with the empty vector or STAT5a vector and MCF7/DOX cells transfected with scramble siRNA or STAT5a-targeting siRNA under treatment with the indicated concentration of DOX determined by Western blotting. ns, p > 0.05; *p < 0.05; **p < 0.01; ***p < 0.001; ****p < 0.0001.

The authors apologize for this error and state that this does not change the scientific conclusions of the article in any way. The original article has been updated.

## Publisher’s Note

All claims expressed in this article are solely those of the authors and do not necessarily represent those of their affiliated organizations, or those of the publisher, the editors and the reviewers. Any product that may be evaluated in this article, or claim that may be made by its manufacturer, is not guaranteed or endorsed by the publisher.

